# Acquired hemophagocytic lymphohistiocytosis as initial manifestation of multiple myeloma: A case report and literature review

**DOI:** 10.1097/MD.0000000000022299

**Published:** 2020-09-25

**Authors:** Fernanda Rodrigues Mendes, Karine Marques Sobral, Hebert Fabricio Culler, Samuel Campanelli Freitas Couto, Juliana Pereira, Vanderson Rocha, Gracia Aparecida Martinez, Luís Alberto de Pádua Covas Lage

**Affiliations:** a Department of Hematology, Hemotherapy and Cell Therapy, Medicine School, Sao Paulo University (FMUSP); b Fundação Pró-Sangue – Hemocentro de São Paulo, São Paulo, Brazil; c Hematology & Hemotherapy, Churchill Hospital, Oxford University, Oxford, UK.

**Keywords:** acute liver failure, hemophagocytic lymphohistiocytosis, multiple myeloma, prognosis, treatment

## Abstract

**Introduction:**

Hemophagocytic lymphohistiocytosis (HLH) is a condition characterized by a hyperinflammatory state and persistent macrophage activation, resulting in reactive phagocytosis of the hematopoietic elements. In children, it is usually a hereditary disorder, while in adults it is usually acquired secondary to viral infections, collagenoses, or tumors. Although accounting for 10% of hematologic malignancies, HLH is rarely associated with multiple myeloma (MM) and other plasmacytic dyscrasias.

**Patient concerns:**

A 64-year-old Brazilian man seeked medical care with a 3-month history of intermittent fever, weight loss, night sweats, and progressive anemic symptoms.

**Diagnosis:**

Total blood count showed severe bicytopenia (normocytic-normochromic anemia and thrombocytopenia), biochemical exams showed elevation of creatinine, as well as monoclonal peak in serum protein electrophoresis, high IgA dosage, and serum immunofixation with IgA kappa paraprotein. Bone marrow biopsy showed 30% of monoclonal and phenotypically anomalous plasmocytes, confirming the diagnosis of MM. Diagnosis of HLH was established by the presence of clinical and laboratory criteria: fever, splenomegaly, cytopenias, hypofibrinogenemia, hyperferritinemia, elevation of triglycerides, and several figures of erythrophagocytosis in bone marrow aspirate.

**Interventions:**

The patient experienced pulse therapy with methylprednisolone for hemophagocytic lymphohistiocytosis, followed by initial therapy for multiple myeloma with cyclophosphamide and dexamethasone.

**Outcomes:**

Once the diagnosis of MM and secondary hemophagocytic syndrome was established, the patient had a rapid clinical deterioration despite the established therapeutic measures, evolving with cardiovascular failure, acute liver failure, acute disseminated intravascular coagulation, worsening renal dysfunction requiring dialysis support, respiratory dysfunction, and lowering of consciousness, characterizing rapid multiple organ dysfunction, ultimately leading to the death of the patient.

**Innovation:**

Here, we aimed to describe the sixth reported case of HLH associated with MM, according to cases cataloged in the PubMed database, and the first case evaluated by 18-fluordeoxyglucose positron emission tomography (18-FDG-PETCT).

**Conclusion:**

Our case report seeks to provide support for a better clinical and laboratory characterization of this rare paraneoplastic entity associated with MM, and aims to call the attention of hematologists and intensivists to this condition that falls within the scope of the differential diagnosis of rapid onset multiple organ failure in patients with plasmacytic neoplasms.

## 1 Introduction

Hemophagocytic lymphohistiocytosis (HLH), also known as hemophagocytic syndrome (HPS) is a potentially life-threatening condition characterized by reactive phagocytosis of the hematopoietic elements.^[1]^ It is marked by persistent activation of CD8 + cytotoxic T lymphocytes and natural killer (NK) cells, leading to increased secretion of inflammatory cytokines and macrophage activation.^[2]^ Macrophages and cytotoxic T cells, via tumor necrosis factor alpha (TNF-alpha) and interferon-gamma stimulation invade the reticuloendothelial system and trigger a cytokine storm.^[3]^ As a hyperinflammatory state, HLH causes signs and symptoms that may progress to multiorgan dysfunction and death.^[2]^

HLH was initially recognized as a hereditary disease that preferentially affected children until the early 1980s, when it was observed that acquired factors could also trigger this phenomenon. Therefore, 2 main forms are recognized, the primary one, that is usually found in children and associated with autosomal recessive mutations in perforin receptor 1 gene (PFR-1)/perforin, protein unc-13 homolog D (UNC13D), or syntaxin 11 gene (STX11) genes. On the other hand, the secondary or acquired HLH occurs in adolescents and adults and is associated with immune dysfunction and malignancies, viral infections such as Epstein-Barr virus and cytomegalovirus, as well bacterial, fungi, or parasitic infection, autoimmune diseases like as systemic lupus erythematosus and rheumatoid arthritis, pregnancy, transplantation, and cytotoxic therapy.^[1,4]^

Malignancies, particularly hematological tumors, account for 45% to 50% of HLH in adults, and result from immune activation by neoplastic cells or loss of inhibitory cell functions mediated by tumor.^[5,6]^ In a recent review article, Vick et al^[7]^ reported 822 cases of cancer-induced HLH. The most common neoplastic cause was aggressive lymphomas (80%) and 46% of them was related to T-cell lymphoma, 28% with B-cell lymphoma, and 6% with Hodgkin lymphoma. Ninety-seven per cent were associated with hematologic neoplasms and 3% of cases were caused by solid tumors.^[7]^

Although plasma cell dyscrasias correspond to 10% of hematologic malignancies, representing 1.8% of all new cancer cases in the United States in 2019 according to Surveillance, Epidemiology and End Results Program (SEER),^[8]^ malignancy-induced HLH were associated with plasma cell neoplasms in only 0.36% of cases.^[7]^ Moreover, there is scarce data in the medical literature regarding this entity associated with multiple myeloma and only a few cases have been reported.^[1,4,9,10,11]^

Hence, here we describe a case of HLH as initial manifestation of multiple myeloma in a Brazilian man who had rapid clinical deterioration with liver, cardio-respiratory, and renal dysfunction associated with coagulopathy and rapidly evolution to death. This report may contribute to a better clinical and laboratory characterization of this rare paraneoplastic manifestation in patients with multiple myeloma.

## 2 Case report

A 64-year-old Brazilian man was admitted to the emergency department of our institution in April 2019 claiming fatigue, malaise, intermittent fever, and weight loss. In the first physical exam he presented hypotension and mental confusion. Three months before he began with progressive prostration, unintentional weight loss of 15 kg, intermittent fever, and night sweats. He was evaluated at an external hematology clinic 2 weeks before to come at our service and was diagnosed with multiple myeloma based on laboratory tests and bone marrow biopsy.

At initial physical examination, he had a temperature of 36.3 °C, a pulse of 81 beats per minute, a respiratory rate of 16 incursions per minute, a peripheral oxygen saturation of 94% in ambient air, a blood pressure of 86/51 mmHg, and a slower peripheral perfusion (4 seconds). He had significant skin-mucous pallor, jaundice 2+/4+, lowered level of consciousness (Glasgow 14), mental confusion, and flapping in the upper extremities. The rest of the physical examination showed no other significant changes.

Initial laboratory evaluation revealed: hemoglobin (Hb) 9.7 g/L, hematocrit (Ht) 28.2%, mean corpuscular volume (MCV) 82.7 fl, white blood count (WBC) 3.02 × 10^9^/L, neutrophils: 2.3 × 10^9^/L, lymphocytes: 0.5 × 10^9^/L, platelets: 39 × 10^9^/L, Na++ 127 mEq/L, K+ 4.3 mEq/L, total Ca++ 9.3 mg/L, P 3.1 mg/L, creatinine 1.84 mg/dL, BUN 122 mg/dL, lactate dehydrogenase (LDH) 648 U/L, and C-protein reactive (CPR) 58.1 mg/L. Hepatic profile showed alanine aminotransferase (ALT) 258 U/L, aspartate aminotransferase (AST) 213 U/L, alkaline phosphatase 386 U/L, gamma GT 473 U/L, total bilirubin: 2.95 mg dL (direct bilirubin [DB]: 2.29 /indirect bilirubin [IB]: 0.66), albumin 2.4 g/dL, prothrombin time/international normalized ratio (PT/INR): 1.27, and activated partial thromboplastin time (aPTT) ratio = 1.23. Laboratory evolution throughout hospitalization is summarized in Table 1.

**Table 1:**
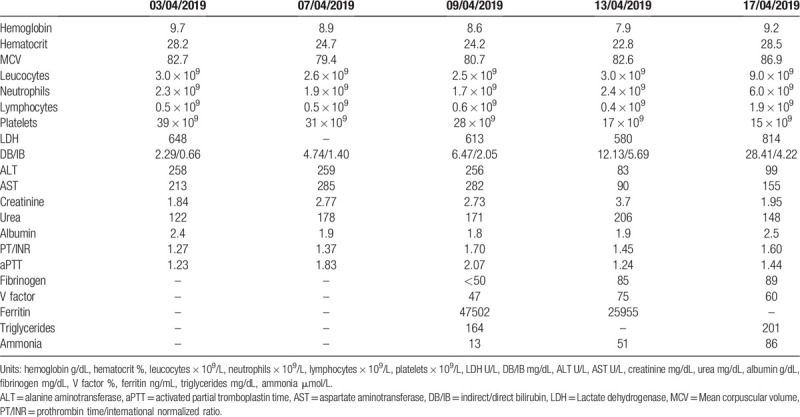
Laboratory evolution during hospitalization.

In the emergency department, peripheral blood and urine cultures were collected and intravenous saline fluid and ceftriaxone was administered for treatment of supposed infectious etiologies. After initial measurements, blood pressure was normalized and the patient was stabilized. In his previous medical history, he had systemic arterial hypertension, congestive heart failure, and chronic renal failure caused by recurrent nephrolithiasis. He used carvedilol 25 mg twice daily, spironolactone 25 mg once daily, ivabradine 5 mg twice daily, AAS 100 mg daily, and rosuvastatin 10 mg daily.

The patient had external laboratory tests showing 0.8 g/dL of monoclonal protein, IgA kappa paraprotein at serum immunofixation, serum IgA of 1038 mg/dL (70–400 mg/dL), and β2 microglobulin of 20.61 mg/L (0.8–2.2 mg/L). Bone marrow biopsy was hypercellular with monoclonal plasma cell proliferation CD56 (+), kappa light chain restriction, representing 30% of the total nucleated cells. A diagnosis of multiple myeloma (MM) Durie Salmon IIIA, ISS III was established in this moment.

**Figure 1:**
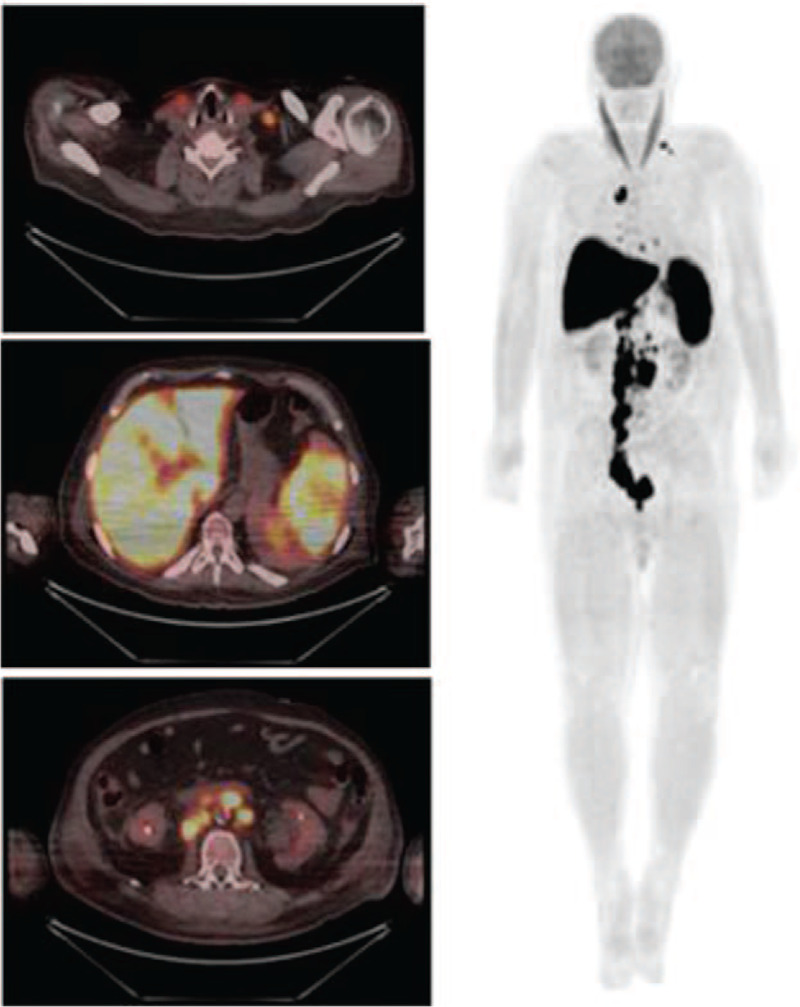
Enhanced 18-FDG uptake in cervical, retroperitoneal lymph nodes, spleen, and liver (SUV max 15.8 in spleen /liver). 18-FDG = 18-fluordeoxyglucose; SUV = standardized uptake value.

Chest, abdomen, and pelvic computerized tomography (CT) revealed multiple lymphadenopathies up to 5.0 cm and splenomegaly. Positron emission CT with 18-fluoro deoxyglucose (18-FDG-PETCT) showed multiple lymphadenopathies in the neck, chest, abdomen, and pelvis with SUVmax 12.0, liver FDG uptake 15.8 (SUVmax), and 16.4 cm splenomegaly with 15.8 spleen FDG uptake (Fig. 1). Echocardiography was negative for endocarditis.

There was negativity for viral hepatitis B and C serology as well as for HIV, HTLV1, chickenpox, toxoplasmosis, herpes, cytomegalovirus, adenovirus, Epstein-Barr virus, parvovirus B19, enterovirus, paraecovirus, and *Mycobacterium tuberculosis.* Blood cultures were negative. As the patient persisted with fever, ceftriaxone introduced at admission was replaced by Piperacillin/Tazobactam and Teicoplanin. However, the patient evolved quickly with multiorgan dysfunction characterized by liver and renal failure, LDH increase, and coagulopathy characterized by prolonged prothrombin time (PT) and activated partial thromboplastin time (aPTT), fibrinogen, and factor V reduction keeping factor VIII level between the normal range values (Table 1).

**Figure 2:**
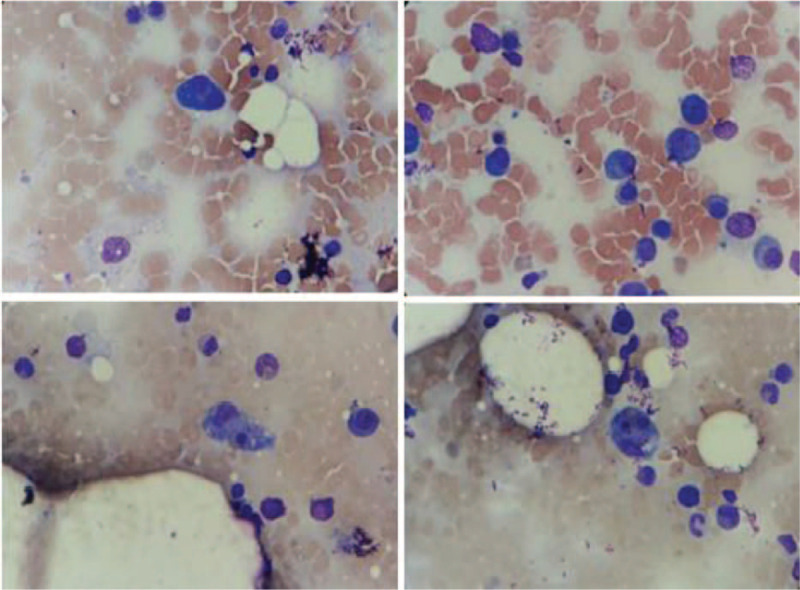
Bone marrow aspirate (40× optic microscopy)—anomalous plasma cell proliferation is observed, with the presence of bi- and trinucleated forms and intense macrophage activity with eritrophagocytosis pictures.

As the clinician suspected of concurrent HLH further tests showed serum ferritin level of 47.502 ng/mL (30–400 ng/mL) and slight elevation of triglycerides. A new bone marrow aspiration confirmed the presence of 21.6% of aberrant plasma cells with significant macrophage activity with several haemophagocytosis pictures (Fig. 2). Immunophenotyping demonstrated 4.9% of anomalous and monoclonal plasma cells expressing CD45^dim^, CD19^partial^, CD38^bright^, CD138^bright^, CD56^dim^, CD117^dim^, and kappa light chain restriction in cytoplasm. Thereafter, the patient was immediately transferred to the intensive care unit and pulse therapy with methylprednisolone 1 g/d for 3 days was administered to control HLH. Afterwards the patient received cyclophosphamide at dose of 500 mg once weekly and dexamethasone 20 mg once daily D1–D4 as anti-multiple myeloma treatment. We were not able to investigate lymphadenopathy because the patient became soon hemodynamically unstable, requiring mechanical ventilation support and renal replacement therapy with hemodialysis. Unfortunately, the patient died 15 days after hospitalization.

## 3 Discussion

Herein we report an uncommon clinical case of MM that present an early and unusual complication characterized by hemophagocytic lymphohistiocytosis. Until now only 6 cases have been described at literature.^[1,4,9,10,11]^ In our service among 1206 MM cases that we have seem in the last 25 years only this case presented HLH as initial manifestation. The patient's relatives provided an informed consent for publication, because he died rapidly after the hospitalization in our service.

In fact, in the clinical practice the HLH diagnosis is usually a challenge because it clinical findings may overcome with a lot of differential diagnoses, as well its laboratory features are far from unspecific. Although secondary HLH has become a medical concern in recent decades, its diagnostic criteria is still based on pediatric studies. However, the HLH 2004 trial included patients above and below aged of 18-year-old seeking for appropriate therapeutic approaches. The HLH was established based in both molecular data and clinical-laboratory features. For HLH diagnosis at least 5 of 8 signals and symptoms must be presented by patients: fever, splenomegaly, cytopenias at least 2 lineages in peripheral blood (Hb < 10.0 g/dL, neutrophils < 1.0 × 10^9^/L, and/or platelets < 100 × 10^9^/L), hypertriglyceridemia (>3.0 mmol/L) or hypofibrinogenemia (<1.5 g/L), hemophagocytosis pictures in bone marrow, spleen or lymph node, lacking or low NK-cell activity, hyperferritinemia (>500 mg/L), and soluble CD25 (soluble IL-2 receptor) >2400 U/mL.^[12]^ Regardless of differences in pathophysiology, etiology, and prevalence of signals and symptoms, the diagnostic criteria proposed in this study are used for diagnosis of HLH in adults and for secondary HLH. Additionally, demonstration of low/lack NK-cell activity and soluble CD25 levels represent a barrier to the diagnosis of HLH and do not add important information in daily clinical practice. In addition, these tests are usually reserved in the setting of research and are not widely available.

Thereafter, on the other hand, it is important to emphasize the differences between HLH manifestations in children and adults. Nikiforow and Berliner^[13]^ has reported that in adults the most prevalent signals are not included in the most used diagnostic criteria for HLH such as the elevation of transaminases that occur in 84% of patients, as well hypoalbuminemia in 92%, high level of LDH in 93%, and acute renal failure in 52%. Fulminant hepatitis followed by acute liver failure like that presented by our patient may be part of this syndrome and has been also reported for other authors.^[14–16]^

Nikiforow and Berliner^[13]^ also highlight the poor correlation between reduced/lack of NK function and secondary HLH, as well as the lack of specificity of ferritin level above 10,000 ng/mL in adults with HLH (60% for adults VS 86–96% for children). Similarly, Schram et al^[6]^ retrospectively identified 113 adults with ferritin levels above 50,000 ng/mL in which only 19% met diagnostic criteria for HLH. They concluded that hyperferritinemia was not able to predict HLH diagnosis in this population.

In our case, the patient met at least 6 of 8 HLH criteria according to 2004 HLH trial. However, we were not able measure the NK cell activity neither the level of soluble CD25. In addition, the patient also showed increment of serum transaminases and LDH, hypoalbuminemia, and acute renal failure suggesting the relevance of other laboratory abnormalities for the diagnosis of this life-threatening syndrome.

Regarding malignancy-associated HLH therapy, there is no standard approach in the medical literature and only rare cases have been prospectively analyzed. Thus, management in this scenario is considered an extrapolation of experience from pediatric patients. In our patient, in addition to antibiotics and clinical support, the patient received corticosteroids to control the hyperinflammatory state and cytokine storm. Unfortunately, he was not exposed to other HLH-targeted therapies as he showed rapid clinical deterioration and progressed to multiple organ dysfunction.

Previous case reports of HLH associated with multiple myeloma are quite rare in the literature and difficult to interpret given their heterogeneity and the presence of other concomitant illness. The patients described so far have different MM status such as at diagnosis period, after autologous hematopoietic stem cell transplantation and in the setting of relapsed/refractory disease. Thereafter, different triggers for HLH have been considered related to MM activity or with ASCT. In addition, various therapies for HLH have been used such as steroids alone, steroids plus immunoglobulin, cyclosporine, etoposide, and multidrug therapy for myeloma, including DRV-PACE regimen (dexamethasone, bortezomib, lenalidomide, cisplatin, doxorubicin, cyclophosphamide and etoposide) and DVP-PACE (dexamethasone, bortezomibe, pomalidomide, cisplatin, doxorubicin, cyclophosphamide and etoposide).^[1,4,9,10,11]^

Unlike other cases that have been reported until now, we performed 18-FDG-PET-CT in our patient, and we found FDG uptake in enlarged lymph nodes, spleen, and liver. Unfortunately, we could not perform lymph node biopsy for histopathological analysis due to the patient's rapid clinical deterioration as described above. However, we have supposed that 18-FDG-PET-CT findings may be explained by the macrophage hyperactivation seemed in the reticuloendothelial system organ. Yuan et al^[17]^ conducted a retrospective study in patients with secondary HLH aiming to evaluate the accuracy of 18-FDG-PET-CT to identify the HLH etiology. The authors have concluded that 18-FDG-PET-CT alone are not enough to discriminate the differential diagnosis between infection and neoplastic cause of HLH. Kim et al^[18]^ also demonstrated that hypermetabolic activity in the spleen, bone marrow, and multiple lymph nodes may also occur in non-neoplastic HLH probably because the FDG should have been up taken by activated immune cells such as macrophages, histiocytes, and T lymphocytes. The only study with a positive correlation between SUV level and underlying cause of secondary HLH was performed by Wang et al.^[19]^ They showed that lymphoma-associated HLH had higher SUVmax in the spleen, liver, lymph nodes, and bone marrow. Nevertheless, the authors did not include other kind of malignancies in this study.

## 4 Conclusion

In conclusion, HLH should be considered in a context of malignancy including multiple myeloma, particularly in patients with significant pro-inflammatory status and rapid clinical worsening concomitant at multiorgan dysfunction. The suspicion and the diagnosis of HLH must be done early and a specific therapy should be promptly started to control the malignancy disease and to interrupting the patient's evolution into a fatal outcome.

## Author contributions

**Conceptualization:** Juliana Pereira, Luís Alberto de Pádua Covas Lage.

**Data curation:** Fernanda Rodrigues Mendes, Karine Marques Sobral.

**Formal analysis:** Hebert Fabricio Culler, Samuel Campanelli Freitas Couto, Gracia Aparecida Martinez.

**Funding acquisition:** Juliana Pereira, Luís Alberto de Pádua Covas Lage.

**Investigation:** Fernanda Rodrigues Mendes, Karine Marques Sobral.

**Methodology:** Gracia Aparecida Martinez, Juliana Pereira, Luís Alberto de Pádua Covas Lage.

**Project administration:** Vanderson Rocha, Juliana Pereira, Luís Alberto de Pádua Covas Lage.

**Resources:** Fernanda Rodrigues Mendes, Karine Marques Sobral.

**Supervision:** Gracia Aparecida Martinez, Juliana Pereira, Luís Alberto de Pádua Covas Lage.

**Validation:** Vanderson Rocha, Juliana Pereira, Luís Alberto de Pádua Covas Lage.

**Writing – original draft:** Fernanda Rodrigues Mendes, Karine Marques Sobral.

**Writing – review & editing:** Gracia Aparecida Martinez, Vanderson Rocha, Juliana Pereira, Luís Alberto de Pádua Covas Lage.
